# Thrombin Assessment on Nanostructured Label-Free Aptamer-Based Sensors: A Mapping Investigation via Surface-Enhanced Raman Spectroscopy

**DOI:** 10.1155/2018/5293672

**Published:** 2018-03-19

**Authors:** Elisa Scatena, Sara Pascale, Cristina Cairone, Filippo Fabbri, Costantino Del Gaudio

**Affiliations:** ^1^Research Consortium Hypatia, Via del Politecnico, 00133 Rome, Italy; ^2^IMEM-CNR, Parco Area delle Scienze 37/A, 43124 Parma, Italy

## Abstract

Aptamers, synthetic single-stranded DNA or RNA molecules, can be regarded as a valuable improvement to develop novel ad hoc sensors to diagnose several clinical pathologies. Their intrinsic potential is related to the high specificity and sensitivity to the selected target biomarkers, being capable of detecting very low concentrations and thus allowing an early diagnosis of a possible disease. This kind of probe can be usefully integrated into a number of different devices in order to provide a reliable acquisition of the analyte and properly elaborate the related signal. The study presents the fabrication and characterization of a label-free aptamer sensor designed using a gold-coated silicon nanostructured substrate to map the target molecule by means of surface-enhanced Raman spectroscopy (SERS). As a proof, thrombin was used as a model at four different concentrations (i.e., 0.0873, 0.873, 8.73, and 87.3 nM). SERS mapping analysis was carried out considering each representative band of the aptamer-thrombin complex (centered at 822, 1140, and 1558 cm^−1^) and then combining them in order to acquire a comprehensive and unambiguous measure of the target. In both cases, a valuable correlation was evaluated, even if the first approach can suffer from some limitations in the third band related to lower definition of the characteristic peak compared to those in the other two bands.

## 1. Introduction

The development of diagnostic devices with high selectivity and specificity is a real demanding clinical need. Particularly in the oncological field, the possibility of dealing with suitable means capable of supporting an early diagnosis implies the definition of ad hoc therapeutic protocols that can promote a valuable outcome [1–3]. Oncological pathologies represent a typical example, being a dramatic clinical and social issue to be addressed as 14.1 million cases around the world were estimated in 2012 and this number is expected to increase to 24 million by 2035 (https://wcrf.org). It is undeniable that designing novel sensors with enhanced sensitivity can effectively support a real advancement in clinics, where the outcome of a pharmacological treatment can be improved if readily administered. However, this potential benefit could be counterbalanced by higher costs related to each production stage that can hamper the wide diffusion of such devices. In addition, these sensors should be also tailored on the specific target molecules to be detected and this might further limit their introduction in the medical practice.

In this regard, aptamers represent a possible alternative that can effectively support this approach, thanks to their high affinity for a specific marker. Aptamers are synthetic, short single-stranded oligonucleotides that fold up into unique 3D structures to specifically bind to selected molecules. They were first reported by Ellington and Szostak [4] and Tuerk and Gold [5] and since then they have been generated against a wide variety of targets, including small molecules [6], peptides [7], amino acids [8], and cell membrane proteins [9]. These molecules are different from antibodies because they not only derive from an in vitro process, but also are prone to bind functional domains of the target protein in specific substrate binding or allosteric sites, thereby modulating the biological function of the molecule [10]. Moreover, they are nonimmunogenic (the chemical production process is not affected by viral or bacteria contamination) and retain their binding and inhibitory behavior even after immobilization on a carrier material [11]. Aptamers are produced by means of an in vitro process named SELEX (Systematic Evolution of Ligands by EXponential enrichment) in which a random oligonucleotide library pool (DNA or RNA) is incubated with the target of interest, the pool containing between 1 · 10^13^ and 1 · 10^15^ members. The SELEX process is based on the ability of these small oligonucleotides to fold in a 3D configuration that exclusively interact with a target by means of Van der Waals surface contact or hydrogen bonding. Generally, many rounds of SELEX are required to isolate aptamers with the highest selective affinity to the target and often a negative selection with a control target is required. The obtained sequences are amplified by PCR (for DNA aptamers) or reverse transcription-PCR (for RNA aptamers). The process is repeated until the pool is enriched for the sequences that specifically recognize the target; finally, the enriched pool is cloned and then sequenced to obtain the individual aptamer of interest. A complete SELEX process ranges from 8 to 16 cycles [12].

In this framework, it should be clearly underlined that although dealing with aptamers to design a sensor is a strategic option to prepare a very sensitive detector, the reliable implementation is critical. Although aptamer-based sensors have been extensively used for the detection of microbial and viral pathogens by means of several procedures, paving the way to the possible development of a clear diagnostic tool for clinics [13, 14], a number of experimental approaches are currently based on the introduction of a label that could be easily detectable and correlated to the concentration of the biomarker to be measured [15, 16]. Clearly, this overcomes the possible limitations related to the reliable use of a label-free sensor, which is definitely the real goal that should be reached to univocally assess the actual potential of aptamer-based detection devices.

To measure very low concentrations of a specific target, surface-enhanced Raman spectroscopy (SERS) is a powerful vibrational spectroscopy technique based on the amplification of electromagnetic fields generated by the excitation of localized surface plasmons of metals nanostructures like gold, silver, and copper. Depending on the structure of the supporting plasmonic material, electromagnetic enhancement for SERS can be theoretically calculated to reach factors of ~10^10^–10^11^, allowing detecting single molecules due to the surface plasmon coupling effect (hotspot) [17, 18]. SERS approach is well-suited for label-free biosensing, mostly if supported by an appropriate data analysis to limit the interference of nonspecific binding [19]. Recently, SERS-based immunoassay has been developed for the detection of pancreas cancer biomarkers, revealing a successful screening among patients with different pathologies [20].

The study here presented, therefore, focused on the definition of an aptamer-based nanostructured sensor sensitive to thrombin, one of the most used models for biosensing. Thrombin is an important regulator in the coagulation cascade and is also implicated in a number of related cancer effects, like tumor growth, metastasis, and angiogenesis, as reviewed by Nierodzik and Karpatkin [21].

The detection analysis was carried out by means of SERS to improve the capability of the proposed device to measure very low concentrations of label-free thrombin and its distribution on the substrate as a result of a surface analysis.

## 2. Materials and Methods

### 2.1. Materials

SERS nanostructured Si (freestanding vertical nanopillars) substrates coated with gold were purchased from Silmeco (Copenhagen, Denmark). Gold elliptical nanoparticles at the top of nanopillars range from 150 to 200 nm; the average size allows evaluating the localized surface plasmon resonance (LSPR) longitudinal mode around 780 nm.

Thiolated thrombin-binding aptamer 5′-HS-(CH_2_)_5_-CH_2_-D/GGT TGG TGT GGT TGG-3′ (TBA15), IDTE buffer nuclease free (10 mM Tris, pH 8.0, 0.1 mM EDTA) for initial resuspension and storage of DNA oligos, and nuclease free water were purchased from Integrated DNA Technologies (Coralville, Iowa, USA).

Tris(2-carboxyethyl)phosphine hydrochloride solution (TCEP), 6-Mercapto-1-Hexanol (MCH), thrombin human recombinant (THR), aqueous solution (≥95%), trizma base, tris(hydroxymethyl)aminomethane (≥99.9%), potassium chloride (99.0%), and magnesium chloride were purchased from Sigma-Aldrich (Milan, Italy).

Hydrochloric acid (37%) and ethanol absolute anhydrous were purchased from Carlo Erba (Milan, Italy).

Phosphate-buffered saline (PBS) 10x, pH 7.4, was purchased from SGM (Rome, Italy).

Samples and solutions were treated with low-binding supplies.

### 2.2. Sensor Preparation

SERS substrates were cleaned by immersion in a solution of HCl 1.5% in ethanol for 2 min and then rinsed with nuclease free water.

In order to unfold the sequence strands and make thiol groups available for the TBA15 immobilization reaction, a thermal treatment at 95°C for 2 min was carried out, followed by a thermal shock in an ice bath for 3 min. TBA15 was treated with a reducing agent, that is, TCEP, in a concentration 100x with respect to the aptamer for 2 h at room temperature in dark conditions.

Sensors were prepared by incubating 5 *μ*M TBA15 solution in PBS for 1 h, rinsed with PBS, and then passivated in 2 mM MCH in PBS for 2 h at room temperature in dark conditions.

Sensors were incubated with thrombin solution at four different concentrations (i.e., 0.0873, 0.873, 8.73, and 87.3 nM) for 1 h at room temperature in dark conditions. Subsequently, substrates were rinsed with THR-buffer and nuclease free water to prevent salt aggregation and allowed to dry.

Before preparation, gold-coated nanopillars are vertically oriented and highly packed, after TBA15-THR complex formation and solvent evaporation, nanopillars lean toward each other, due to the surface tension during the drying process, to induce hotspots formation thanks to the coupling effect of LSPRs between adjacent nanostructures.

### 2.3. SERS Analysis and Processing of Collected Data

SERS measurements were carried out using an InVia Qontor Raman Microscope (Renishaw, UK). The excitation source was a 785 nm He-Ne laser, the wavelength was chosen in order to obtain a large SERS amplification due to resonant conditions. Five different regions (100 *μ*m × 120 *μ*m each) on the nanostructured gold substrates were mapped to collect 12000 spectra (a total amount of 60000 spectra were acquired for each sensor) with a step size of 1 × 1 *μ*m setting the following configuration: 50x objective, 0.3 mW laser power, and 1 s acquisition time. Spectra resolution was 2 cm^−1^.

The aptamer-thrombin complex was assessed considering the three main characteristic Raman peaks previously reported, that is, 822, 1140, and 1558 cm^−1^ [22]. Data were exported from the WiRE software (Renishaw, UK), after cosmic ray removal, baseline correction, and integration within a spectral range of ±20 cm^−1^ centered on the Raman shift values above reported, similarly to Yang et al. [23].

Two different approaches were considered for data processing by means of a custom-made script (Matlab, The MathWorks, Natick, Ma, USA). In the first one, the three main bands were independently processed in order to assess the individual sensitivity to thrombin detection. For this aim, all the values larger than 60% of the maximum one measured over the five investigated areas were considered and normalized with respect to that value in order to avoid the influence of a possible intensity fluctuation among nanostructured sensors. A representative index was then calculated as the sum of all these processed values and the coefficient of variation of the resulting distribution for each thrombin concentration was evaluated.

In the second approach, the characteristic bands were not independently considered in order to perform a synthetic analysis of the sensors and taking into account the thrombin “fingerprint” in a single step. For this aim, a comprehensive matrix of each investigated area was obtained as the cube root of the product of the maps associated with the three typical Raman shift values. Then, the same processing procedure above reported was applied to this data set.

In both cases, a curve fitting of the relationship between normalized integral intensities and thrombin concentrations was subsequently performed.

Measurements were carried out in triplicate for each thrombin concentration.

Referring to the characterization of the TBA15-quadruplex, Raman spectra were recorded using the drop coating deposition method, similarly to Pagba et al. [24]. Briefly, 100 *μ*l of 5 *μ*M oligonucleotide in PBS solution was deposited on nanostructured surface for 1 h and the surface was passivated with 2 mM MCH for 2 h. Subsequently, the sample was soaked in a solution of 100 mM KCl for 1 h to allow the quadruplex folding of the oligonucleotide structures. Before Raman analysis, the substrate was rinsed with nanopure water and dried at room temperature to favour nanopillars leaning.

## 3. Results and Discussion

Aptamers can be a valuable means for the detection of specific biomarkers. Their high affinity and selectivity to the target can effectively contribute to improve the diagnostic and therapeutic protocols introducing label-free methodologies. When this characteristic is coupled with ad hoc measurement techniques, it is then possible to deal with an experimental targeted setup capable of quantitatively collecting the presence of the biomarker at very low concentrations. For this aim, SERS was selected here for the detection of thrombin bound to thiolated aptamers on functionalized gold nanopillars.

**Figure 1:**
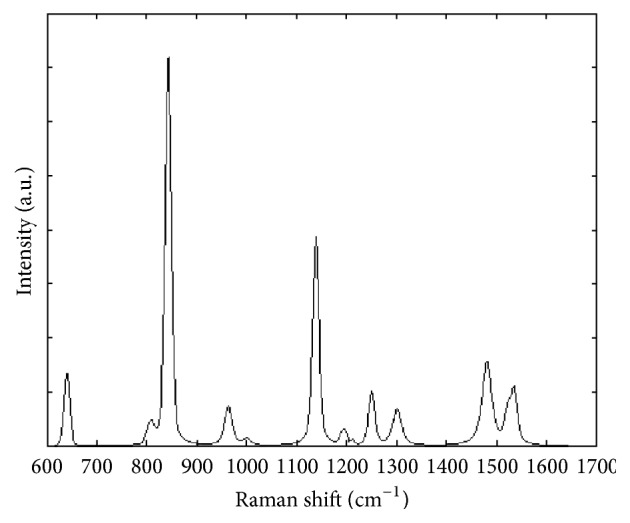
SERS spectrum of the aptamer-thrombin at 87.3 nM.

**Figure 2:**
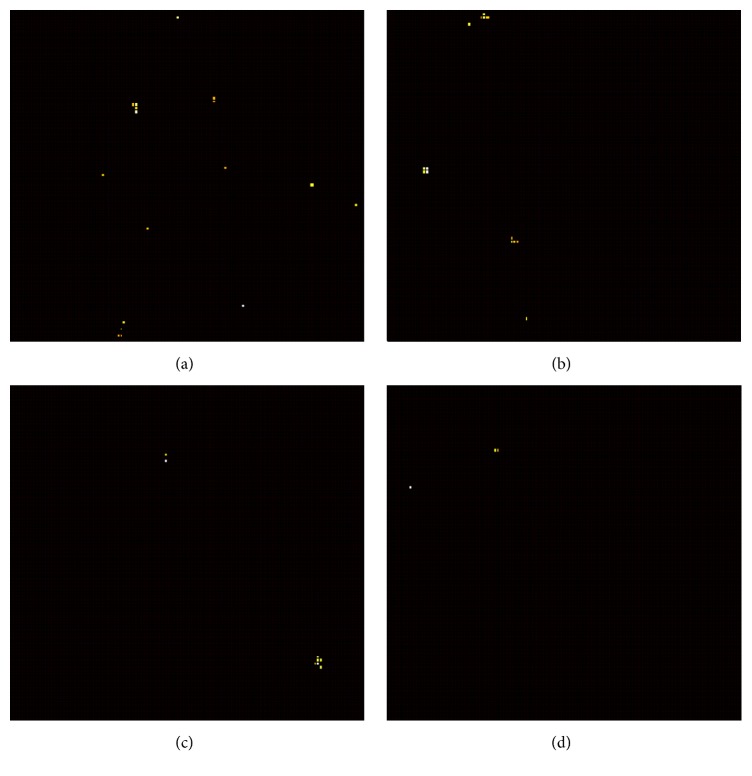
SERS mapping distribution of the thrombin-aptamer complex for the four investigated protein concentrations: (a) 87.3 nM; (b) 8.73 nM; (c) 0.873 nM; (d) 0.0873 nM.

A typical Raman spectrum of the aptamer-thrombin complex is shown in Figure 1. An example of the relationship between the aptamer-thrombin complex with thrombin concentration, in terms of number of detected pixels, is reported in Figure 2. This result clearly displays that increasing the protein concentration leads to a more abundant hotspot detection and, in turn, support the proposed technical approach to sum the detected intensities as a measurement index for protein quantification.

**Figure 3:**
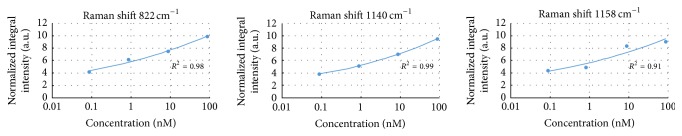
Power fitting curves for the relationship SERS intensity versus thrombin concentration for each of the three characteristic bands.

Following the first analytical method a relevant correlation was computed including all the data collected from the sensors for each vibrational frequency (Figure 3), especially for spectral ranges centered at 822 and 1140 cm^−1^, which can be referred to the combined C2′-endo and C3′-endo modes of the 2′-deoxyribose sugars and to the C–O–C stretch [22]. Conversely, a slightly less accurate result was observed for the band at 1558 cm^−1^, the guanine ring modes [22], that could be ascribed to a broader shape of the band of this vibrational frequency (Table 1 summarizes the related coefficients of variations for each spectral band). Even if the three peaks are representative of the aptamer-thrombin complex, this approach implies that their informative content is independently considered and the individual diagnostic relevance can be limited, while a single and homogenous output would be desirable. Such a consideration can be stressed further when a label-free measurement is performed as a reliable detection is of paramount importance and, therefore, the identification of an indisputable “fingerprint” of the analyzed protein is mandatory. In this regard, a direct evaluation based on the detection of each single peak should be carefully treated because it cannot assure a univocal determination. It should be also taken into account that the sensitivity of each spectral range can be different for the investigated concentrations, thus concurring to a not uniform performance of the sensor. Considering this framework, it is then reasonably to assume that a more consistent measure can be obtained if single maps are combined together to assess a unique information which automatically includes each spectral contribution in a quantitative sense, following the analytical methodology proposed here for the collected results.

**Figure 4:**
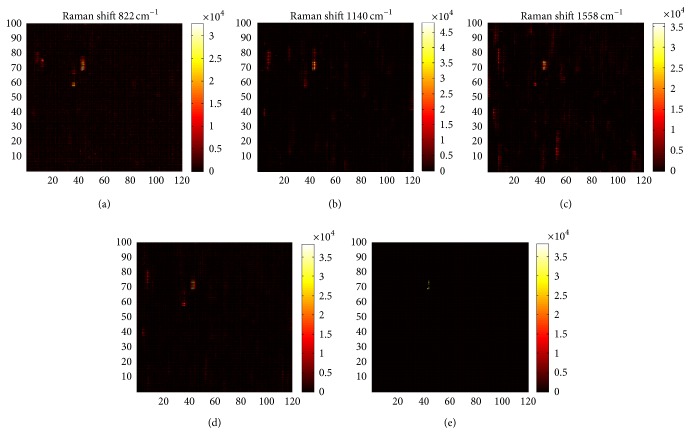
Regional mapping of the SERS substrate for 87.3 nM thrombin concentration. (a), (b), and (c) show the individual Raman maps for the characteristic bands, while (d) reports the combined map and (e) reports the resulting one after applying the threshold of 60%.

**Figure 5:**
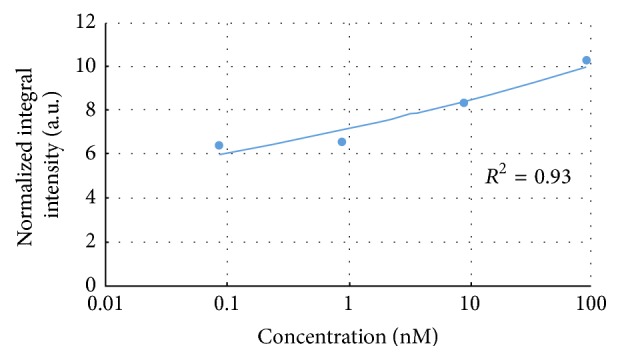
Power fitting curve for the relationship SERS intensity versus thrombin concentration for the three characteristic bands simultaneously considered.

The investigation carried out according to the second method aimed to evaluate the incidence of the three bands simultaneously considered. This approach offers a synthetic measure of the response of the aptamer-thrombin complex to the different target concentrations.  Figure 4 shows the acquired map of each single band ((a), (b), and (c)), the combined map, and the resulting one obtained by applying the threshold level of 60% ((d) and (e)). The regression analysis, including again all the data collected from the sensors, highlighted a remarkable fitting, demonstrating a strong relationship between SERS measurements and thrombin concentration (Figure 5). Similarly, to the previous case, Table 2 summarizes the coefficients of variation for the investigated distribution.

This combined method is extremely tailored to elaborate only those regions where a valuable SERS signal is detected, cutting out spectra that could negatively affect the final measurement. Moreover, the visual inspection of the map at 1558 cm^−1^ readily confirms why the investigation focused only on this band can lead to data collection in which the characteristics of the acquired signal should be carefully considered before to carry out the subsequent analysis. Compared to the maps at 822 and 1140 cm^−1^, a less defined detection can be observed, being strictly related to the shape of the peak and its intensity (refer to Figure 1 where sharper peaks are clearly detectable in the first two ranges). However, it should be underlined that a setup refinement has to be considered in the future development. Even if a valuable correlation between SERS signals and thrombin concentrations was verified, coefficients of variations are not uniformly distributed within the range of thrombin concentration and this might be referred to the intrinsic lack of control on the hotspot formation, their distribution, and the size of the assembled nanopillar clusters on the substrate surface.

The investigation presented here is based on the quantitative detection of a label-free biomarker, differently from a number of reports mainly focused on this method of analysis, also involving the design of a specific detection and measurement protocol [25–27]. Clearly, the latter one can easily support the recognition of the target, but it implies additional manipulations of the sample before evaluation as labelling biomolecules is a time-consuming procedure and can imply the loss of biological activity [24]. Moreover, considering the complex nature of biological fluids, it is crucial to select a specific binding molecule that univocally tags the analyte of interest. Even if this procedure can offer a reliable detection, also introduce a number of experimental steps to properly prepare the sample that could not be promptly translated to a clinical laboratory.

In addition, from a technical point of view, SERS assays based on nanoparticles can be characterized by a potential drawback due to their characteristics in terms of size, shape, crystallinity, and overall morphology [28]. It should be also considered that synthesized measuring nanoprobes are typically suspended in solutions and this can be disadvantageous because (i) they can precipitate and thus move away from the laser focal point; (ii) only those irradiated by the laser are measured, being a small percentage which reduces the probed volume; (iii) and the lack of reference points in solution can introduce an additional limitation to precisely focus on the sample for SERS measurement [29]. Therefore, the use of a large SERS-active surface, as the one here considered, could represent an improvement, even if a number of issues can be listed as well, as previously reported.

**Figure 6:**
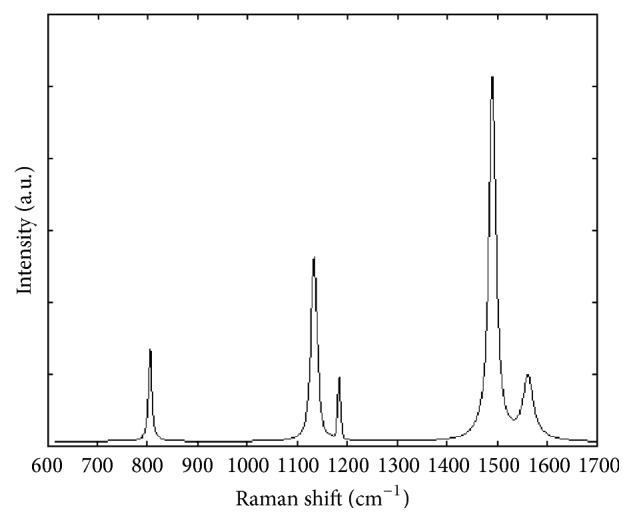
SERS spectrum of the TBA15-quadruplex at 5 *μ*M.

In order to further assess the reliability of the proposed approach, Raman spectra of the quadruplex thrombin-binding aptamers were acquired in absence of thrombin. The quadruplex structure is restored due to the stabilizing effect of K^+^ ions as highlighted by the presence of the characteristic peak at 1480 cm^−1^ (Figure 6) [24]. The TBA is a well characterized chair-like, antiparallel quadruplex structure that binds specifically to thrombin and consists of two G-tetrads connected by three edge-wise loops: two TT loops (T^3^T^4^ and T^12^T^13^) at one end and a single T^7^G^8^T^9^ loop at the other end. The conformational distribution of the four coplanar 2′-deoxyguanosine in the G-quartets of the TBA aptamer is well defined and is stabilized by cyclic Hoogsteen hydrogen bounding [30]. Comparing this spectrum to that reported in Figure 1, the specific response of the aptamer to thrombin underlined by the three characteristic peaks of the complex formation can be easily highlighted.

## 4. Conclusions

Labelled detection is a straightforward technical methodology, but it implies a manipulation of the biological sample that might limit its routinely application in clinics. To overcame this possible drawback, this study presented the SERS mapping assessment of label-free aptamer-thrombin complex on nanostructured substrates as a valuable approach for diagnostic purposes. Four different thrombin concentrations were considered, in a range of 0.1–100 nM, finding a significant correlation. Further improvements are needed in order to test the method on biological samples acquired in physiological or pathological conditions to actually assess the potential of the proposed technique. If SERS “fingerprint” of a specific biomarker is known, then a reliable label-free measurement can be integrated into clinical practice to offer highly sensitive tools for early diagnosis.

## Figures and Tables

**Table 1:** Coefficients of variation for each spectral band considered in the SERS analysis for the four investigated thrombin concentrations.

Thrombin concentrations [nM]	Raman shift [cm^−1^]		
	822	1140	1558
0.0873	0.3422	0.1773	0.1867
0.873	0.0454	0.0859	0.0451
8.73	0.1894	0.2749	0.0759
87.3	0.0913	0.1526	0.1755

**Table 2:** Coefficients of variation for the combined SERS mapping for the four investigated thrombin concentrations.

Thrombin concentrations [nM]	
0.0873	0.0385
0.873	0.0922
8.73	0.1961
87.3	0.1872
